# The Italian Version of the Difficulties in Emotion Regulation Scale-8 (DERS-8): A Two-Step Assessment of Structural Validity, Psychometric Properties, and Clinical Cut-Off

**DOI:** 10.1186/s40359-025-03482-6

**Published:** 2025-10-14

**Authors:** Alessandro Alberto Rossi, Anna Panzeri, Stefania Mannarini

**Affiliations:** 1 Department of Philosophy, Sociology, Education, and Applied Psychology, Section of Applied Psychology, University of Padova, Padova, Italy; 2 Center for intervention and Research on Family studies – CIRF, Department of Philosophy, Sociology, Education, and Applied Psychology, Section of Applied Psychology, University of Padova, Padova, Italy; 3 Department of General Psychology, University of Padova, Padova, Italy

**Keywords:** Emotion dysregulation, Difficulties in emotion regulation, DERS, Confirmatory factor analysis, Measurement invariance, cut-off scores

## Abstract

**Background:**

Emotion regulation difficulties (ERD) serve as a transdiagnostic risk factor for psychopathology, and the Difficulties in Emotion Regulation Scale (DERS) is a widely-used self-report measure. Brief versions of such tools are essential for integration into longer assessment batteries and for longitudinal monitoring. However, DERS-8 has not yet been validated in the Italian context.

**Objective:**

This study aimed to provide the Italian validation of DERS-8 by examining its psychometric properties, factorial structure, and cut-off scores to distinguish between acceptable and at-risk levels of ERD, along with normative data.

**Methods:**

In Study 1 (*N* = 2016), confirmatory factor analysis (CFA) was used to test the DERS-8 structural validity. Also, its psychometric properties were tested. In Study 2 (*N* = 4221), measurement invariance across age and gender was conducted in an independent sample, together with a deep investigation of psychometric properties and clinical cut-off.

**Results:**

Study 1 revealed that, initial model fit was poor but improved to good fit indexes after correlating specific residual pairs. Indeed, after correlating residuals between items #2-#4, #1-#7, and #3-#6, IT-DERS-8 provided good fit indexes (RMSEA = 0.042; 90%CI[0.033, 0.052], CFI = 0.997; SRMR = 0.029) as well as good psychometric properties. Study 2 successfully replicated these results and showed that it is invariant between groups male and females and between *≤* 49 and ≥ 50 years old. Lastly, a cut-off of 25 showed adequate accuracy in classifying individuals with ‘non-problematic’ and ‘at risk’ ERD (AUC = 0.864).

**Discussion:**

The IT-DERS-8 has solid psychometric properties and, given its normative data and cut-off, represents a valid measure for assessing and identifying individuals with non-problematic (vs. at risk) in Italian adult samples.

**Supplementary Information:**

The online version contains supplementary material available at 10.1186/s40359-025-03482-6.

## Introduction

In recent years, there has been a growing interest in emotional regulation [1–3], which is now recognized as a crucial aspect of healthy psychological functioning [4]. The significant influence of emotions on personal experiences, thoughts, and behaviors is well-documented [5, 6]. Emotions, on one hand, are temporary states tied to specific situations and can have either positive or negative valence [3]. Conversely, emotional regulation can be understood as a multifaceted concept that encompasses a range of skills aimed at comprehending and managing emotions and feelings, including efforts to affect emotions and their outward expression [4, 7]. Emotion regulation difficulties (ERD) refer to challenges in effectively managing and responding to emotional experiences, often involving problems with accepting emotions, controlling impulses during distress, and accessing adaptive regulation strategies [2, 7, 8].

Emotion regulation is a common underlying factor linked to various psychological conditions across the life span. ERD, also known as emotion dysregulation [7] can have significant consequences for clinical practice. For example, issues with emotion regulation may increase subjective distress and exacerbate impulsivity, potentially resulting in maladaptive behaviors such as emotional eating, self-harm, and substance abuse [8, 9]. By triggering or aggravating clinical issues, ERD serves as a predictor of psychopathology [10–12] and can profoundly impact life [13, 14]. In fact, ERD play a role in the onset and persistence of mental health disorders, thereby acting as a transdiagnostic vulnerability factor [9] underlying various psychopathological conditions and contributing to comorbidities [15, 16]. These conditions include mood and anxiety disorders, eating disorders, attention deficit/hyperactivity disorder, conduct issues, substance abuse, and psychotic disorders [15].

Moreover, ERD are strongly linked to psychological distress [17, 18], insomnia [19], and impulsivity [20, 21]. Additionally, research has indicated that ERD are among the vulnerability factors associated with anxiety and depression [22, 23], eating disorders [24, 25], addiction [26], and personality disorders [27, 28]. Given its transdiagnostic characteristics, the emphasis on ERD has become increasingly significant in recent decades [9, 29]. To address this, effective, concise, and precise assessment tools are essential in both clinical and research settings [30, 31].

Currently, the Difficulties in Emotion Regulation Scale (DERS) [7] is recognized as one of the most prevalent tools for evaluating ERD in both adults and adolescents globally [30]. The DERS has undergone validation in multiple countries, including the United States, Canada, the United Kingdom, Germany, Spain, and Italy [32–39].

The DERS is a self-report questionnaire with 36 items and a six-factor structure, addressing how individuals who experience emotions of sadness and frustration respond to and regulate them. Its six dimensions include: (a) Non-acceptance of Emotional Responses, which refers to the tendency to reject emotions and develop negative secondary emotional reactions like denial and distress; (b) Difficulties Engaging in Goal-Directed Behavior, which involves challenges in completing tasks and maintaining focus when experiencing negative emotions; (c) Impulse Control Difficulties, which pertain to behavioral issues when experiencing negative emotions; (d) Lack of Emotional Awareness, which includes struggles in recognizing, attending to, and acknowledging emotions; (e) Limited Access to Emotion Regulation Strategies, stemming from beliefs about the inability to manage and regulate emotions; (f) Lack of Emotional Clarity, which involves difficulties in identifying and understanding the emotions being experienced, leading to confusion. The DERS subscales show significant positive correlations with symptoms of psychopathology [30], despite some evidence showed that the dimension of lack of awareness may not represent the same overarching emotion regulation construct [40].

Moreover, due to its effectiveness, the DERS is extensively utilized across various psychological environments, both in clinical settings and among the general population. In the general population, the DERS has been employed throughout different life stages [41], including during adolescence [42, 43], parenthood [44], and in relation to attentional [45] and executive functions (e.g., memory) [46]. The DERS has also found significant application within clinical populations [47–49], particularly among individuals with anxiety disorders [50], severe mental health issues [51], as well as those facing physical illnesses and chronic conditions [52–54]. To reiterate DERS’s importance, numerous studies have underscored the correlation between DERS scores and symptoms of borderline personality disorder in both clinical and non-clinical groups [47], as well as its link to internet addiction in young adults over a year [55].

The original DERS long version comprises 36 items and does not always facilitate a quick and routine evaluation of ERD. The administration can be cumbersome, complicating its inclusion in extensive assessment batteries [31]. Consequently, Kaufman et al. developed the DERS Short Form (DERS-SF), then adapted in several countries including Italy [30, 31, 56, 57].

Given the widespread use of the DERS in both clinical and research environments, and its relevance to studying and treating various clinical disorders [49, 58], there is a clear benefit to developing an even more concise version of the measure. This would be valuable for multiple stakeholders interested in assessing ERD. Moreover, another reason for shortening the DERS relies in persistent psychometric issues with the Lack of Awareness subscale that performs poorly, has weak internal consistency and weak or absent associations with other subscales and indices of psychopathology [49, 59]. This subscale appears to assess a construct – emotional noticing or awareness – that is conceptually distinct from other facets of emotion regulation, which mostly focus on how individuals respond to and manage emotions rather than simply noticing them [40]. Excluding the Awareness subscale from shortened versions, improves internal consistency and construct validity, supporting the use of shorter forms that focus more specifically on ERD rather than emotional awareness per se [59].

While the DERS is widely used to assess ERD, some researchers have noted challenges in its ability to clearly differentiate between normative variations and ERD [49, 60]. This limitation partly arises from the broad distribution of DERS scores often observed in non-clinical samples, which may reflect a wide range of typical regulatory experiences rather than exclusively pathological difficulties [49]. Additionally, the DERS focuses on the presence of ERD but places less emphasis on the functional impact or frequency of these difficulties in daily life, which are critical for distinguishing clinically meaningful impairment [61]. These considerations underscore the need for continued research to refine the interpretation and clinical utility of DERS-based measures, including brief forms.

Moreover, recently there has been a growing demand for extremely concise and effective versions of assessment tools in both clinical and research settings [62–64], which maintain precision and construct validity. These brief instruments can reduce the burden on respondents, minimize redundancy, lower research costs, and facilitate easy monitoring of responses to interventions in clinical settings, ultimately enhancing data quality. Typically, tracking patient outcomes involves using a set of short measures [65].

The Difficulties in Emotion Regulation Scale-8 (DERS-8) [66] was recently proposed as a concise self-report version derived from the original 36-item DERS, developed to efficiently assess overall ERD among both adolescents and adults while improving psychometric properties by excluding problematic subscales. The DERS-8’s items cover difficulties in acceptance, goal-directed behavior under distress, impulse control, and access to effective emotion regulation strategies, with two items per dimension. Unlike the DERS-SF, which typically retains multiple subscales reflecting distinct facets of emotion regulation (e.g., Nonacceptance, Goals, Strategies, Clarity, and Awareness in various versions), the DERS-8 prioritizes brevity and a focused construct representation [66]. Specifically, the DERS-8 excludes two subscales from the original DERS-36 that are retained in many DERS-SF versions: Awareness and Clarity. The Awareness subscale has been consistently found to have psychometric issues, including low internal consistency and weak correlations with other subscales, suggesting it measures a construct conceptually distinct from core ERD [40]. Excluding Awareness and Clarity leads to a more internally consistent unidimensional scale in the DERS-8, but at the cost of some conceptual breadth related to emotional awareness and understanding [49].

The DERS-8 is particularly useful as a self-report tool for identifying ERD [67] – although cut-off scores identifying individuals with adequate/non-problematic and inadequate ERD (i.e., problematic/at risk) have never been provided. An increasing number of studies supports the unidimensional factorial structure of the DERS-8 with eight items [49, 66–69], each beginning with the phrase “When I’m upset, …” such as “When I’m upset, I have difficulty getting work done.” This phrasing helps respondents focus on situations where managing negative emotions is necessary, thereby enabling the measurement of emotion regulation based on an individual’s ability to engage in adaptive behaviors when experiencing negative feelings [7].

Prior validation studies have demonstrated the DERS-8’s robust internal consistency, unidimensional factor structure, and meaningful construct validity across clinical and nonclinical populations, including adolescents with psychiatric disorders and older adults [66, 67, 70–72]. Its brevity and psychometric coherence enhance its practical utility in research and initial screening. However, the exclusion of Awareness and Clarity subscales narrows the conceptual breadth, potentially omitting important facets of emotional awareness and understanding [49]. Additionally, existing studies have focused mainly on construct validity and measurement invariance, with limited validation against clinical diagnostic criteria or sensitivity to treatment outcomes [67, 69]. Therefore, while the DERS-8 offers a psychometrically sound and efficient tool, further research is needed to clarify its clinical utility and how well it captures the full complexity of ERD compared to the full DERS.

Studies have also shown the DERS-8 effectiveness in diverse populations, such as Polish adults, where it serves as a reliable predictor of mental health outcomes [49, 66, 67, 69, 70].

However, the psychometric validation of the Italian version of DERS-8 is still lacking, despite its usefulness for clinical and research aims. Given the aforementioned reasons and the cross-cultural variability of the DERS, it is crucial to conduct a thorough validation and examination of the DERS-8 psychometric properties in the Italian context. A concise tool like this would be more appropriate for inclusion in longer assessment batteries or surveys, where minimizing the number of items is essential to reduce assessment time for respondents or to evaluate multiple constructs within the same timeframe [73]. Additionally, in healthcare settings such as hospitals or clinics, shorter assessment tools are preferred since patients may have limited attention spans or may become fatigued more easily.

The objective of this research was twofold. Study 1 sought to investigate the structural validity and convergent validity of the Italian version of DERS-8 (IT-DERS-8) using confirmatory factor analysis (CFA). In an independent sample, Study 2 aimed to cross-validate the factorial structure of the IT-DERS-8 and test its measurement invariance across sex and age, and developing a possible clinical cut-off, while also providing normative data.

## Study 1: Assessing factorial structure and psychometric properties of the IT-DERS-8

### Methods and materials

#### Sample size determination

The sample size was planned a priori by means of the “*n: q* criterion” – a subject-per-parameter ratio criterion – where *n* is number of subjects and *q* is the number of model parameters to be estimated [74]. A minimum of 400 participants was ensured, maintaining a ratio of 10 subjects per parameter (10 subjects per 40 parameters; n_min_ = 400).

#### Procedure

In line with the original development and validation study of the scale, the items of the DERS-8 were extracted from the already existing version of the DERS, which is widely validated and used in Italian as well [38]. For this reason, the items already available in Italian were used [56].

An online survey was created in Qualtrics and the anonymous link was posted on social media platforms (e.g., Facebook, Twitter/X). Snowball sampling [75, 76] was used to recruit individuals from a convenience sample of the general population. Inclusion criteria comprised: (A) age over 18, (B) proficiency as a native Italian speaker, (C) completion of the assessment procedure (D) provide informed consent, (E) completion time less than 10 or greater than 20 min [77].

A pilot study using an online questionnaire featuring the IT-DERS-8 and other questionnaires was conducted with 20 participants (excluded from subsequent statistical evaluations). This preliminary assessment aimed to verify item clarity and establish a reasonable completion timeframe, between 10 and 20 min. According to recent guidelines, before data analysis, a screening to control quality data for online survey was performed [78]. Also, in line with inclusion criteria, participants with any missing responses to IT-DERS-8 items were excluded from analyses. No item-level imputation was performed, and only complete cases were retained for all analyses.

All participants voluntarily consented to take part in the study and signed informed consent. The study was in accordance with the Ethical standards of the Ethics Committee of the University of Padua (protocol n° 5402, approved: 10th of June 2023).

#### Participants

An initial sample of 2194 participants was contacted, 178 questionnaires were excluded due to inappropriate completion times (< 10 or > 20 min; *n* = 73) and/or missing data/answers (*n* = 105). The final sample included 2016 participants: 433 males (21.5%) and 1583 females (78.5%), aged from 18 to 83 y.o. (*mean* = 41.58, *SD* = 12.682). Table 1 reports a more detailed sample analysis.

**Table 1:** Study 1 and Study2. Samples’ descriptive statistics

	Study 1 (*N* = 2016)			Study 2 (*N* = 4221)	
Age (*mean*,* SD*)	41.58	12.68		43.03	12.62
Minimum-maximum	18	83		18	84
Sex (*n*, %)					
Male	433	21.5%		870	20.6%
Female	1583	78.5%		3351	79.4%
Education (*n*, %)					
Middle school	523	25.9%		859	20.4%
High school	828	41.1%		1703	40.3%
Bachelor	583	28.9%		1453	34.4%
Master degree/ PhD	82	4.1%		206	4.9%
Work status (*n*, %)					
Student	128	6.3%		200	4.7%
Full-time worker	1153	57.2%		2494	59.1%
Entrepreneur	348	17.3%		752	17.8%
Part-time worker	144	7.1%		241	5.7%
Unemployed	155	7.7%		311	7.4%
Retired	88	4.4%		223	5.3%
Civil status (*n*, %)					
Single	285	14.1%		591	14.0%
In a relationships	591	29.3%		1160	27.5%
Married	902	44.7%		1904	45.1%
Separated	145	7.2%		323	7.7%
Divorced	72	3.6%		189	4.5%
Widowed	21	1.0%		54	1.3%

#### Measures

An information form was used to collect general demographic information (i.e., sex, age, civil status, education level, and employment status).

##### Difficulties in emotion regulation Scale – 8 (DERS-8)

The Italian Difficulties in Emotion Regulation Scale – 8 (IT-DERS-8) [66] represents an ultra-short 8-item adaptation of the original DERS [7], designed to efficiently assess ERD while maintaining psychometric robustness. Unlike the DERS original version and its short version (DERS-SF) [56], this 8-item version assesses a single first-order dimension, on which all items load, defined as ‘ERD.’ In line with its previous versions, the DERS-8 maintains a 5-point Likert response scale, from 1 (= *“almost never”*) to 5 (= *“almost always*”). There are no reverse-scored items. As in previous versions, higher scores correspond to greater difficulties in managing emotions. The final version of the IT-DERS-8 is reported in Supplementary Material 1 and Supplementary Materials 2 (Table S1).

##### Toronto alexithymia scale 20 (TAS-20)

The TAS-20 [79] is the predominant questionnaire utilized for evaluating alexithymia, a deficiency in the cognitive processing of emotions [80]. TAS-20 demonstrated robust psychometric qualities in both clinical and nonclinical populations [81]. The TAS-20 evaluates three primary aspects of alexithymia: Difficulty Identifying Feelings (DIF), Difficulty Describing Feelings (DDF), and Externally Oriented Thinking (EOT). It is based on 20 items evaluated using a 5-point Likert scale, ranging from 1 (“strongly disagree”) to 5 (“strongly agree”). Elevated scores signify increased challenges in cognitive emotion processing. The Italian version of the TAS-20 was used [82]. The questionnaire, in its original Italian validation, showed good psychometric properties, with a satisfying factorial structure [82] an high internal consistency and test-retest reliability. In this study, the TAS-20 and its subscales show adequate internal consistency: DIF: McDonald’s ω = 0.848; DDF: McDonald’s ω = 0.732; EOT: McDonald’s ω = 0.642; and Total Score: McDonald’s ω = 0.859.

##### Emotion regulation questionnaire (ERQ)

The ERQ [83] is a 10-item self-report instrument that evaluates two techniques of emotion regulation: ‘cognitive reappraisal’ (CogR) and ‘expressive suppression’ (Supp.). The ERQ uses a 7-point Likert scale, with responses ranging from 1 (“strongly disagree”) to 7 (“strongly agree”). Elevated scores signify increased utilization of that emotion regulation strategy. The ERQ demonstrated robust psychometric qualities in both clinical and nonclinical populations. The Italian version of the ERQ was used [84]. The questionnaire, in its original Italian validation, showed good psychometric properties, with an adequate factorial structure [84] and high internal consistency and adequate convergent validity. In this study the two dimensions of the ERQ proved adequate internal consistency: Cog.R.: McDonald’s ω = 0.820 and Supp. : McDonald’s ω = 708.

##### The Barratt impulsiveness scale (BIS-11)

The BIS-11 [85] is a questionnaires assessing the presence of impulsive behaviors and traits. The BIS-11 evaluates impulsivity based on the concept that it is a construct composed of different facets, such as attentional impulsivity (i.e., lack of cognitive persistence), motor impulsivity (i.e., acting on impulse), and non-planning impulsivity (i.e., lack of a sense of the future). However, numerous studies have shown that these factors do not appear to be independently usable, and the literature largely agrees in defining the scale as unidimensional [86]. The questionnaire consists of 30 items with a 4-point Likert response scale (ranging from 1 = *“never”* to 4 = *“always”*). In the present study, the Italian version of the BIS-11 [86] was used. The questionnaire, in its original Italian validation, showed good psychometric properties, with a satisfying factorial structure [86] and high internal consistency and adequate convergent validity. In the present study, the Italian version of the BIS-11 demonstrated a good internal consistency: McDonald’s ω = 0.807.

#### Statistical analyses

Statistical analyses were conducted using the R software (v 4.3.2) and the following packages: lavaan [87], psych [88], and tidyverse [89].

As a preliminary analysis, item distribution (e.g., skewness, kurtosis) were inspected to guide the estimator choice, moreover items’ correlations were examined rule out the presence of excessively relationships (possible presence of multicollinearity) which could bias the factorial analysis results (*r* should be < |0.84|) [90–92].

To confirm the factorial structure of the IT-DERS-8, a confirmatory factor analysis (CFA) was conducted, specifying a one-factor model. Specifically, all eight items of the scale loaded onto a single first-order factor. Given the response scale, the diagonally weighted least squares (DWLS) estimator was used [93]. The DWLS is particularly advised for categorical or Likert-type data, as it yields more precise parameter estimates and standard errors than maximum likelihood estimation, which presumes continuous and normally distributed data [74, 93–95].

According to literature, model fit was assessed using classical indices: chi-square statistic (χ^2^), the Root-Mean Square Error of Approximation (RMSEA), the Comparative Fit Index (CFI), and the Standardized Root Mean Residual (SRMR) [74, 93]. The model’s goodness of fit was evaluated based on the following criteria: (A) a non-significant χ^2^ statistic, (B) an RMSEA value below 0.08, (C) a CFI above 0.95, and (D) an SRMR below 0.08 [74, 93, 96].

Furthermore, the items’ capacity to differentiate respondents with low or high levels of the examined construct was evaluated using item discriminant power (IDP) [97, 98]. The highest total score for each scale of the IT-DERS-8 and the quartile rank for each subject were computed in greater detail. Independent sample t-tests were subsequently conducted, along with their effect size (Cohen’s *d*) [99], to evaluate the discriminatory power of the item, utilizing the total score of the scale as the dependent variable and its lowest and highest quartiles as the grouping variable [97, 98]. Additionally, the adjusted item-total correlation (*r*_Adj_.) was calculated [92]. The internal consistency of the IT-DERS-8 was assessed with McDonald’s ω [100] and convergent validity was still assessed with the Pearson correlation coefficient [92] and interpreted using Cohen’s benchmarks [99].

## Results

### Preliminary analysis

Items of the IT-DERS-8 showed an almost univariate normal distribution (Table 2) and none of the bivariate correlations exceeded the critical level of 0.84. Indeed, the highest correlation was observed between item#2 and item#4 (*r* = .641).

**Table 2:** Study 1 and study 2. Item descriptive statistics, item psychometric properties, and confirmatory factor analysis (CFA)

	Descriptive statistics					IDP				CFA	
	Mean	SD	SK	K		*t*	*d*	*rAdj*		|λ*sf*|	*R* ^2^
Study 1											
Item#1	2.35	1.230	0.749	-0.530		-34.64	2.16	0.547		0.586	0.343
Item#2	2.00	1.109	1.149	0.506		-31.78	1.98	0.577		0.607	0.369
Item#3	1.77	1.125	1.466	1.127		-28.17	1.75	0.551		0.622	0.386
Item#4	2.01	1.091	1.165	0.663		-36.22	2.26	0.667		0.722	0.521
Item#5	2.10	1.126	1.004	0.184		-34.99	2.19	0.595		0.725	0.525
Item#6	2.20	1.210	0.887	-0.282		-35.62	2.22	0.556		0.606	0.367
Item#7	2.63	1.223	0.550	-0.837		-45.95	2.88	0.653		0.719	0.517
Item#8	2.22	1.051	0.955	0.357		-35.86	2.24	0.644		0.775	0.600
Study 2											
Item#1	2.33	1.191	0.785	-0.402		-47.89	2.10	0.525		0.549	0.301
Item#2	1.93	1.043	1.249	0.984		-41.58	1.83	0.567		0.599	0.359
Item#3	1.72	1.078	1.600	1.702		-39.04	1.72	0.547		0.612	0.374
Item#4	1.97	1.053	1.211	0.897		-45.79	2.01	0.626		0.667	0.445
Item#5	2.04	1.108	1.096	0.446		-49.37	2.17	0.608		0.756	0.571
Item#6	2.21	1.222	0.912	-0.232		-47.01	2.06	0.531		0.570	0.324
Item#7	2.61	1.181	0.647	-0.654		-64.83	2.84	0.645		0.706	0.498
Item#8	2.22	1.065	0.972	0.360		-48.49	2.13	0.600		0.743	0.552

Notes: SD = Standard deviation; SK = Skewness; K = Kurtosis; IDP *=* Item discriminant power; *t* = *t*-test; *d* = Cohen’s *d* (Effect size); *r*Adj. = item-total correlation (adjusted); λ = standardized factor loading; *R*^2^ = Item explained variance

### Structural validity

Overall, the IT-DERS-8 did not show good fit indices. Indeed, the χ^2^ was statistically significant [χ^2^ (20) = 719.093; *p* < .001] and the RMSEA did not meet the recommended threshold suggesting a non-ideal fit: RMSEA = 0.132 90%CI[0.124; 0.140]; despite a good CFI (0.967) and an adequate SRMR (0.075).

Consequently, in line with previous studies [101], a examination of univariate modification indices was carefully performed [93, 94, 102, 103]. This examination suggested that that the model might be enhanced by connecting the residuals of item#2 and item#4 (Δχ^2^ = 314.545). After rerunning the model, it was still not satisfactory, so the same analysis of univariate modification indices was conducted. This analysis suggested correlating the residuals of item#1 and item#7 (Δχ^2^ = 188.543). Despite these modifications, a final analysis of the univariate modification indices suggested correlating the residuals of item#3 and item#6 (Δχ^2^ = 139.412). A detailed analysis of the items suggested a possible interdependence between these indicators, which could justify the correlation between their residuals (see discussion section). Upon correlating these items residuals, the model exhibited an improved fit to the data (r_item_#2-#4 = 0.504; r_item_#1-#7 = 0.419; r_item_#3-#6 = 0.390) [93, 94, 102, 103]: χ^2^ (17) = 77.242; *p <* .001; RMSEA = 0.042; 90%CI[0.033, 0.052], CFI = 0.997, SRMR = 0.029. As reported in Table 2, all 8 items’ loadings were statistically significant and ranged from 0.586 (item#1) to 0.775 (item#8) with and items’ explained variance raging from 0.343 (item#1) to 600 (item#8).

### Internal consistency and psychometric properties

The IT-DERS-8 showed good internal consistency (McDonald’s ω = 0.856). As reported in Table 2, the IDP analysis showed that all 8 items of the IT-DERS-8 discriminated well between subjects with low or high level of the measured construct. The discrimination parameter *t*_i_ ranged from |28.17| (item#3) to |45.95| (item#7), with an associated effect size (Cohen’s *d*) ranging from 1.75 (large effect) to 2.88 (large effect), respectively. In addition, the item-total correlation (adjusted) revealed strong association between each item and the total score (see Table 2, column r_adj_).

### Correlation between psychometric measures

Table 3 shows the small-to-large correlations among the IT-DERS-8 and the other psychometric measures. A moderate association is observed between the IT-DERS-8 and the alexithymia TAS-20 total score (*r* = .499, *p* < .001). However, when considering the specific DIF (difficulties in identifying feelings) subscale of TAS-20, the association becomes stronger (*r* = .548, *p* < .001). In contrast, statistically significant but weaker associations are observed between the IT-DERS-8 and the other two TAS-20 subscales – DDF (difficulties in describing feelings) (*r* = .352, *p* < .001) and EOT (externally oriented thinking) (*r* = .220, *p* < .001). Additionally, a weak association is found with emotion regulation strategies such as cognitive reappraisal (*r* = − .256, *p* < .001) or emotional suppression (*r* = .108, *p* = .001). Finally, in line with the literature, a moderate correlation is observed between the IT-DERS-8 and the total impulsivity scale measured with the BIS-11 (*r* = .336, *p* < .001).

**Table 3:** Study 1 and study 2. Correlations among variables

	Descriptives					Correlations							
	Mean	SD	SK	K		DERS-8	TAS20	DIF	DDF	EOT	Cog.R.	Supp.	Impuls.
Study 1													
DERS-8	17.28	6.47	0.98	0.44		-							
TAS20	56.49	10.74	0.53	0.17		0.499	-						
DIF	16.01	6.40	0.62	-0.30		0.548	0.880	-					
DDF	12.94	4.59	0.26	-0.64		0.352	0.725	0.591	-				
EOT	18.20	4.86	0.13	-0.20		0.220	0.465	0.356	0.474	-			
Cog.R.	5.05	1.19	-0.42	0.06		− 0.256	− 0.150	− 0.224	− 0.204	− 0.271	-		
Supp.	3.49	1.40	0.29	-0.43		0.108^*^	0.404	0.271	0.487	0.381	− 0.005^§^	-	
Impuls.	60.71	9.90	0.56	0.47		0.336	0.415	0.440	0.201^*^	0.438	− 0.260	− 0.055^§^	-
Study 2													
DERS-8	17.03	6.20	1.02	0.69		-							-
TAS20	55.99	10.44	0.45	-0.19		0.483	-						-
DIF	15.88	6.53	0.55	-0.53		0.566	0.874	-					-
DDF	12.72	4.85	0.36	-0.69		0.342	0.727	0.569	-				-
EOT	17.10	4.71	0.25	-0.43		0.164	0.463	0.327	0.441	-			-
Cog.R.	5.12	1.19	-0.54	0.13		− 0.264	− 0.142	− 0.214	− 0.176	− 0.259	-		-
Supp.	3.35	1.40	0.25	-0.60		0.170	0.450	0.302	0.555	0.365	0.007^§^	-	-

*Notes*: all correlations are statistically significant with *p* < .001; except for: * *p < .*010 and § *p* > .050 *ns.*DERS-8 = Difficulties in Emotion Regulation Scale 8; TAS20 = Toronto Alexithymia Scale. DIF = Difficulty Identifying Feelings. DDF = Difficulty Describing Feelings. EOT = Externally Oriented Thinking.; CogR = ERQ Cognitive reappraisal; Supp = ERQ Suppression; Impuls. = BIS11 – total score

## Study 2. Measurement invariance, psychometric properties and cut-offs of the IT-DERS-8

### Materials and methods

#### Sample size determination

In line with Study 1, the *n: q* criterion was also used in this case. Consequently, the minimum number of participants to be recruited was 400.

#### Procedure

The same procedure of Study 1 was applied and a different sample from study 1 was enrolled. Snowball sampling method [75, 76] was used to recruit participants via Facebook and Twitter. The inclusion criteria were the same of Study 1.

Also in this case, a pilot study with 20 participants (not included in the statistical analysis) tested the online questionnaire, including the IT-DERS-8, to verify item clarity and set a reasonable completion time. The same data quality control procedure used for Study 1 was also applied to this study [78]. Moreover, even in this case, participants with missing responses were excluded from the final sample.

All participants voluntarily consented to take part in the study and signed informed consent forms. The study was in accordance with the ethical standards of the Ethical Committee of the University of Pauda (protocol n° 5402, approved: 10th of June 2023).

#### Participants

An initial sample of 4469 participants completed the survey, but 248 participants were excluded due to inappropriate completion times (< 10 or > 20 min; *n* = 69) or missing answers (*n* = 179). The final sample included 4221 participants: 870 males (20.6%) and 3351 females (79.4%), aged 18 to 84 y.o. (*mean* = 43.03, *SD* = 12.616). A detailed analysis of the sample is reported in Table 1.

#### Measures

The same biographic information form of Study 1 and the IT-DERS-8 was used to collect general demographic information and psychometric measures, respectively. In particular, based on the results of Study 1, the same constructs were investigated, such as alexithymia through the TAS-20 and emotion regulation strategies through the ERQ, in addition to the IT-DERS-8. For the purposes of the study, no questionnaire was administered to measure impulsivity. Again, these questionnaires showed good internal consistency indices: IT-DERS-8: McDonald’s ω = 0.846; TAS*-*total score: McDonald’s ω = 0.858; DIF: McDonald’s ω = 0.816; DDF: McDonald’s ω = 0.777; EOT McDonald’s ω = 0.636; Cog.R: McDonald’s ω = 0.838; Supp.: McDonald’s ω = 0.734.

#### Statistical analysis

The R software was used to perform statistical analysis with the following packages: lavaan [87], psych [88], pROC [104], and tidyverse [89].

In line with Study 1, preliminary analyses were conducted again before performing the main analyses of the study. Subsequently, the factorial structure of the Italian version of the DERS-8 was tested again. In this case as well, the DWLS estimator was used, and the model’s goodness of fit was assessed using the fit indices previously described (i.e., RMSEA, CFI, and SRMR), following their standard cut-off values (see Sect. 2.5) [74, 93, 96]. Once it was established that the factorial structure of the DERS-8 in this study also matched the one found in Study 1, correlation analysis and measurement invariance (MI) analyses were conducted, along with the identification of possible cut-off scores.

To assess whether the factorial structure of the IT-DERS-8 remains consistent across gender (male vs. female) and age (median split: age < 49y.o. vs. >50y.o.), MI analyses were conducted [105–107]. According to guidelines [105, 107], first, the model structure was independently tested for each sample. If the model fits adequately for each group, four nested models are sequentially defined with increasing constraints: the first model tests for configural invariance (constraining to equivalence the factorial structure between groups); the second one tests metric invariance (constraining to equivalence factor loadings); the third evaluates strong invariance (constraining to equivalence item thresholds); and the fourth assesses means invariance (constraining to equivalence latent means between groups) [108]. These models were compared sequentially to evaluate the impact of each additional constraint. Model comparisons were conducted using differences in three fit indices, with specific criteria serving as thresholds for determining model equivalence. These criteria included a chi-square difference (Δχ^2^) with a *p*-value greater than 0.050, a change in the CFI (ΔCFI) less than |0.010|, and a change in the RMSEA (ΔRMSEA) less than |0.015 [93, 109]. Exceeding these thresholds, combined with a poorer model fit, was interpreted as evidence of model inadequacy.

Moreover, through receiver operating characteristic (ROC) curve analysis, cut-off scores were obtained to differentiate individuals with “non-problematic” ERD from those with “at risk” ERD. Since no gold standard is currently available for defining the two groups (i.e., individuals with “non-problematic” vs. “at risk” ERD), the methodology employed in previous studies [110, 111] was used, following a multi-step approach. Specifically, on the basis of correlation results of Study 1 and Study 2, (A) a multivariate latent variable (MLV) was created ad hoc, including measures of ERD symptoms such as the DIF and the DDF from the TAS-20, and the ERQ Cog.R scale. (B) The factor score (FS) of this MLV was extracted [74] and normalized creating a range varying from 0 to 100. (C) Critical points in the FS distribution were selected. Specifically, FS values above the 75th, percentiles were considered indicators of ERD and labeled as “cases” (conversely, FS values below the 75th percentiles were labeled as “controls”) [110, 111]. This procedure was also repeated for the 80th, 85th, 90th, and 95th percentiles of the normalized FS distribution. (D) The overall accuracy-validity of the IT-DERS-8 was estimated using the area under the ROC curve (AUC; 5000 stratified bootstrap samples), interpreted according to Swets’ criteria: AUC = 0.50, null; AUC from 0.51 to 0.70, small; AUC from 0.71 to 0.90, moderate; AUC from 0.91 to 0.99, high; and AUC = 1.00, perfect accuracy [112, 113]. Additionally, sensitivity (Se) and specificity (Sp) were calculated for each cut-off point [114, 115].

Finally, following established methods from prior research [56, 116], based on the approach outlined by Gary et al. in 2021 [117], normative T scores for the IT-DERS-8 were computed, along with distribution percentiles [117, 118].

## Results

### Structural validity

In line with Study 1, the IT-DERS-8 showed an non-ideal fit to the data. Indeed, the χ^2^ was statistically significant [χ^2^ (20) = 2009.502; *p <* .001], the RMSEA and the SRMR did not meet the recommended threshold: RMSEA = 0.154 90%CI[0.148; 0.159]; SRMR = 0.089 – despite an adequate CFI (0.950).

According to Study1, an examination of univariate modification indices suggested to correlate residuals of item#2 and item #4 (Δχ^2^ = 880.953), then residuals of item#1 and item#7 (Δχ^2^ = 516.432), and residuals of item#3 and item#6 (Δχ^2^ = 413.453). Also in this case, upon correlating the residuals of these items, the model exhibited an improved fit to the data (ritem#2-#4 = 0.543; ritem#1-#7 = 0.461; ritem#3-#6 = 0.448) [94, 119–121]. At this point the IT-DERS-8 showed a good fit to the data: χ^2^ (17) = 200.719; *p* < .001; RMSEA = 0.051; 90%CI[0.044, 0.057], CFI = 0.995, SRMR = 0.033.

Table 3 shows that all items’ loadings were statistically significant and ranged from 0.549 (item#1) to 0.756 (item#5) with and items’ explained variance raging from 0.301 (item#1) to 0.571 (item#5).

### Correlation between psychometric measures

In line with Study 1, Table 3 shows the small-to-large correlations among the IT-DERS-8 and the other psychometric measures. A moderate association is observed between the IT-DERS-8 and the DIF subscale (*r* = .566, *p* < .001) and the DDF subscale (*r* = .342, *p* < .001). In contrast, a small association was found between the IT-DERS-8 and EOT subscale (*r* = .164, *p* < .001). Additionally, a small association is found with emotion regulation strategies such as cognitive reappraisal (*r* = − .264, *p* < .001) or emotional suppression (*r* = .170, *p* < .001).

### Measurement invariance

*Gender* (male vs. female). The models for males and females were tested separately and showed good fit indices. This allowed for further statistical analysis (Table 4). The configural invariance analysis demonstrated a good model fit. Next, the metric invariance model was tested, which not only proved to be statistically adequate but also did not show a significant decline in fit indices compared to the previous model [Δχ^2^ (7) = 10.714; *p =* .152; |ΔRMSEA| = 0.004; |ΔCFI| = 0.000]. The analysis then proceeded by testing the equality of thresholds between groups, which also proved to be statistically appropriate. The comparison with the metric invariance model did not indicate a significant decrease in fit [Δχ^2^ (23) = 20.484; *p = .*613; |ΔRMSEA| = 0.009; |ΔCFI| = 0.000]. Finally, latent means invariance was tested, and once again, it was found to be statistically adequate, with no significant decline compared to the previous model [Δχ^2^ (1) = 3.617; *p* = .057; |ΔRMSEA| = 0.000; |ΔCFI| = 0.001]. These results suggest that for the IT-DERS-8, males and females share the same structural model (configural invariance), interpret the items with the same strength (metric invariance), have equivalent item thresholds (strong invariance), and exhibit the same latent mean for the construct (latent means invariance).

**Table 4:** Study 2. Measurement invariance across genders and age

	χ2 (df)	RMSEA	CFI		DIFF-TEST	*p*(DIFFTEST)	|ΔRMSEA|	|ΔCFI|
Gender								
Model ‘Male’ (*n* = 870)	31.136 (17)	0.031	0.998					
Model ‘Female’ (*n* = 3351)	179.136 (17)	0.053	0.995					
Configural Inv.	210.272 (34)	0.050	0.996					
Metric Inv.	220.986 (41)	0.046	0.995		10.714 (7)	0.152	0.004	0.000
Strong Inv.	241.470 (64)	0.036	0.996		20.484 (23)	0.613	0.009	0.000
Mean Inv.	245.087 (65)	0.036	0.995		3.617 (1)	0.057	0.000	0.000
AGE								
Model ‘*≤* 49 y.o.’ (*n* = 2857)	127.136 (17)	0.048	0.996					
Model ‘*≥* 50 y.o.’ (*n* = 1364)	88.079 (17)	0.055	0.994					
Configural Inv.	215.215 (34)	0.050	0.995					
Metric Inv.	258.198 (41)	0.050	0.995		42.983 (7)	< 0.001	0.000	0.001
Strong Inv.	296.647 (64)	0.042	0.994		38.450 (23)	0.023	0.009	0.000
Mean Inv.	439.436 (65)	0.052	0.991		142.790 (1)	< 0.001	0.010	0.004

Note: χ^2^ = chi-square test; *df* = degrees of freedoms; RMSEA = root mean square error of approximation; CFI = comparative fit index; |Δ(…)| = absolute value of the differences between indices

*Age* (*≤* 49 y.o. vs. *≥* 50 y.o.). The models for *≤* 49 y.o. and *≥* 50 y.o. were analyzed separately, both demonstrating strong fit indices, which justified proceeding with further statistical testing (Table 4). The configural invariance analysis confirmed that the overall factor structure was consistent across age bands. Subsequently, the metric invariance model was evaluated, showing statistical adequacy without a meaningful reduction in fit indices compared to the previous model [Δχ^2^ (7) = 42.983; *p* < .001; |ΔRMSEA| = 0.000; |ΔCFI| = 0.001]. The next step involved assessing whether item thresholds were equivalent between groups, which also yielded statistically acceptable results. Comparing this model with the metric invariance model did not indicate a significant drop in fit [Δχ^2^ (23) = 38.450; *p* = .023; |ΔRMSEA| = 0.009; |ΔCFI| = 0.000]. Finally, latent mean invariance was examined, confirming that the model remained statistically robust, with no substantial decrease in fit relative to the previous step [Δχ^2^ (7) = 142.790; *p <* .001; |ΔRMSEA| = 0.010; |ΔCFI| = 0.004]. These findings indicate that *≤* 49 y.o. and *≥* 50 y.o. exhibit an equivalent structural model (configural invariance), interpret the items with the same level of association to the construct (metric invariance), have comparable item thresholds (strong invariance), and share the same latent mean for the measured trait (latent means invariance) in the IT-DERS-8.

### Cut-off score proposals

Given the exploratory nature of the analysis, effect size values (i.e., AUC) are reported here for the different percentile points used as the independent variable in the ROC Curve (75th, 80th, 85th, 90th, and 95th). A more comprehensive analysis of all the values for SE, SP, and ACC is provided in Table 5.

**Table 5:** Cut-offs scores

		75th percentile				80th percentile				85th percentile				90th percentile				95th percentile		
Thr.		SE	SP	ACC		SE	SP	ACC		SE	SP	ACC		SE	SP	ACC		SE	SP	ACC
9		0.995	0.027	0.271		0.995	0.025	0.211		1	0.025	0.163		1	0.023	0.122		1	0.022	0.071
10		0.990	0.059	0.294		0.989	0.055	0.234		0.993	0.053	0.186		0.995	0.051	0.146		0.995	0.049	0.096
11		0.980	0.128	0.343		0.978	0.119	0.284		0.985	0.115	0.238		0.993	0.111	0.200		0.995	0.106	0.150
12		0.962	0.213	0.402		0.958	0.199	0.344		0.966	0.191	0.301		0.981	0.186	0.266		0.995	0.177	0.218
13		0.931	0.319	0.474		0.933	0.301	0.423		0.948	0.290	0.383		0.962	0.281	0.350		0.981	0.269	0.305
14		0.893	0.435	0.551		0.900	0.412	0.506		0.918	0.397	0.470		0.941	0.385	0.441		0.967	0.369	0.399
15		0.856	0.532	0.613		0.867	0.505	0.575		0.888	0.487	0.543		0.925	0.474	0.520		0.962	0.455	0.480
16		0.811	0.606	0.657		0.827	0.578	0.626		0.858	0.560	0.602		0.911	0.547	0.584		0.943	0.524	0.545
17		0.765	0.672	0.696		0.786	0.644	0.672		0.822	0.625	0.653		0.883	0.612	0.639		0.925	0.587	0.604
18		0.698	0.726	0.719		0.727	0.701	0.706		0.774	0.683	0.696		0.843	0.670	0.688		0.892	0.646	0.658
19		0.635	0.774	0.739		0.677	0.753	0.738		0.725	0.736	0.734		0.791	0.722	0.729		0.868	0.699	0.707
20		0.569	0.814	0.752		0.616	0.796	0.761		0.672	0.781	0.765		0.744	0.769	0.766		0.830	0.746	0.750
21		0.507	0.845	0.759		0.548	0.828	0.774		0.603	0.815	0.785		0.681	0.805	0.792		0.778	0.784	0.784
22		0.462	0.873	0.769		0.509	0.859	0.792		0.561	0.846	0.806		0.634	0.836	0.815		0.736	0.816	0.812
23		0.415	0.898	0.776		0.462	0.885	0.804		0.521	0.875	0.825		0.599	0.866	0.839		0.698	0.846	0.839
24		0.368	0.914	0.776		0.411	0.903	0.809		0.464	0.893	0.833		0.533	0.885	0.849		0.646	0.869	0.857
25		0.324	0.931	0.778		0.362	0.921	0.814		0.414	0.913	0.842		0.488	0.907	0.864		0.623	0.893	0.879
26		0.288	0.945	0.779		0.320	0.935	0.817		0.370	0.928	0.849		0.434	0.922	0.873		0.552	0.909	0.891
27		0.248	0.959	0.780		0.274	0.950	0.820		0.328	0.946	0.859		0.397	0.941	0.886		0.519	0.930	0.909
28		0.215	0.970	0.779		0.238	0.962	0.823		0.290	0.958	0.864		0.352	0.954	0.893		0.462	0.944	0.919
29		0.183	0.978	0.778		0.205	0.972	0.824		0.251	0.969	0.867		0.310	0.965	0.899		0.406	0.956	0.928
30		0.146	0.984	0.772		0.165	0.979	0.823		0.209	0.977	0.869		0.258	0.974	0.902		0.358	0.967	0.937
31		0.123	0.989	0.770		0.141	0.985	0.823		0.176	0.983	0.869		0.218	0.981	0.904		0.311	0.975	0.942
32		0.097	0.990	0.765		0.111	0.987	0.819		0.137	0.986	0.866		0.176	0.984	0.903		0.264	0.981	0.945
33		0.073	0.992	0.760		0.085	0.990	0.817		0.109	0.990	0.865		0.141	0.989	0.903		0.222	0.986	0.948
34		0.058	0.995	0.758		0.069	0.993	0.816		0.087	0.993	0.864		0.115	0.992	0.904		0.170	0.989	0.948
35		0.044	0.996	0.756		0.057	0.996	0.816		0.072	0.995	0.865		0.094	0.995	0.904		0.146	0.993	0.950
36		0.034	0.997	0.754		0.044	0.997	0.814		0.057	0.997	0.864		0.073	0.996	0.903		0.108	0.995	0.950
37		0.023	0.998	0.752		0.031	0.999	0.813		0.039	0.998	0.862		0.054	0.998	0.903		0.080	0.997	0.951
38		0.013	0.999	0.750		0.017	0.999	0.811		0.020	0.999	0.860		0.028	0.999	0.901		0.038	0.998	0.950
39		0.008	0.999	0.749		0.010	0.999	0.810		0.012	0.999	0.860		0.016	0.999	0.900		0.024	0.999	0.950

Note: Thr = Threshold; SE = Sensitivity; SP = Specificity; ACC = Accuracy

**Fig. 1:**
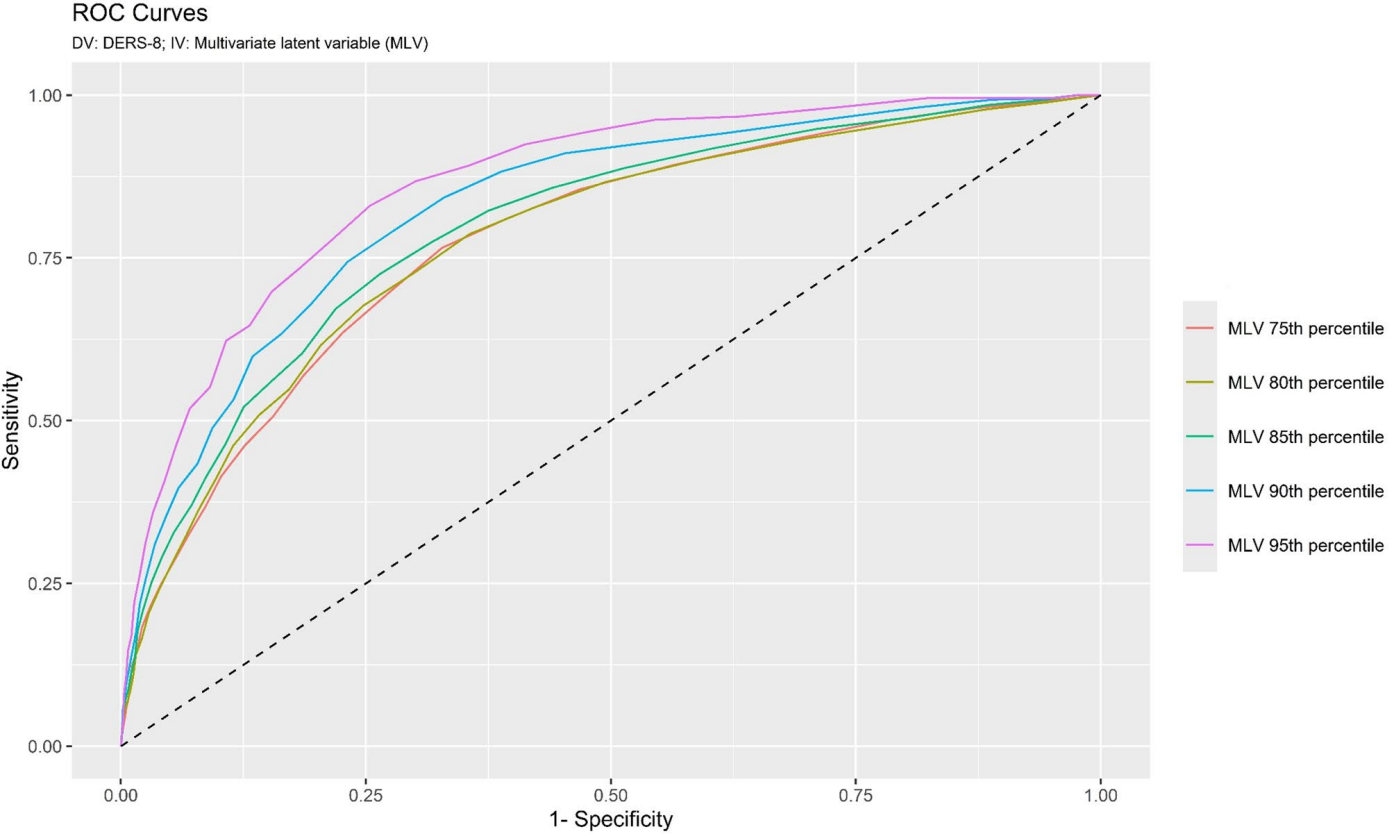
ROC curves cut-offs

Using the 75th percentile of the MLV distribution as a critical threshold to distinguish individuals with “non-problematic” ERD from those “at risk”, the IT-DERS-8 demonstrated a low/moderate accuracy in differentiating between these groups: AUC = 0.777, se = 0.008 (95%CI[0.761; 0.793]); *p* < .001 (Fig. 1; red line). Using the 80th percentile of the MLV distribution, the IT-DERS-8 demonstrated a moderate accuracy in differentiating between these groups: AUC = 0.778, se = 0.008 (95%CI[0.760; 0.796]); *p* < .001 (Fig. 1; brown line). Using the 85th percentile of the MLV distribution, the IT-DERS-8 demonstrated a low/moderate accuracy in differentiating between these groups: AUC = 0.796, se = 0.010 (95%CI[ 0.777; 0.816]); *p* < .001 (Fig. 1; green line). Using the 90th percentile of the MLV distribution as a critical threshold to distinguish individuals with “non-problematic” ERD from those “at risk”, the IT-DERS-8 demonstrated a moderate accuracy in differentiating between these groups: AUC = 0.829, se = 0.010 (95%CI[ 0.809; 0.849]); *p* < .001 (Fig. 1; blue line). Using the 95th percentile of the MLV distribution as a critical threshold to distinguish individuals with “non-problematic” ERD from those “at risk” the IT-DERS-8 demonstrated a moderate/high accuracy in differentiating between these groups: AUC = 0.864, se = 0.012 (95%CI[ 0.839; 0.888]); *p* < .001 (Fig. 1; purple line). Consequently, the analysis was further explored, yielding the higher AUC.

By using the 95th percentile of the MLV distribution as the gold standard, considering a cut-off score of 25 (i.e., IT-DERS-8 ≥ 25: at-risk individual), the ROC curves showed a ACC of 0.879 with a SE of 0.623 (95%CI[0.557; 0.689]), and a SP of 0.893 (95%CI[0.883; 0.902]). Moreover, 86.69% of individuals were classified as having adequate/non-problematic ERD, while 13.31% were considered at risk (total sample = 4221). Therefore, using the reported cut-off of 25 for the IT-DERS-8 scale, the ROC curves indicated that 84.79% of individuals were correctly classified as “true negatives” and 3.13% as “true positives” (87.92% correct classifications). Conversely, 1.90% were classified as “false negatives” and 10.19% as “false positives” (12.09% incorrect classifications).

### Normative scores

On the sample of 6237 adults from the Italian general population (sample of Study 1 merged with Study 2), both the normative scores (T-score: mean = 50, *SD* = 10) and the percentile distribution of the IT-DERS-8 were calculated. Results are reported in in the Supplementary Materials 2 (Table S2).

## Discussions

Considering that ERD are common across various psychological disorders and play a crucial role in psychological well-being, there was a need for a concise and effective assessment tool applicable in both clinical and research environments to minimize assessment duration, alleviating respondents fatigue during surveys and facilitating the integration of brief tools into multi-construct and extensive assessment frameworks in both clinical and research contexts. Since the Italian version of DERS-8 had not been validated, the primary aim of this research was to validate the IT-DERS-8 by examining its factorial structure and exploring its psychometric properties through two studies conducted in two independent samples, involving adults from the Italian general population. Importantly, cut-offs were proposed to differentiate adequate and at-risk levels of ERD.

### Study 1

Study 1 showed that the IT-DERS-8 has a unidimensional factor structure, in accordance with the foreign validations, and each item significantly loaded onto the dimension. However, the DERS-8 shows some issues if residual correlations are not modeled. The most significant concern is an unacceptable RMSEA. While this is not necessarily problematic, it does warrant statistical attention because before making any modifications to the model, the authors thoroughly examined the potential changes in terms of item content. Items #2 (“I feel out of control”) and #4 (“I have difficulty controlling my behaviors”) both relate to difficulties or loss of control. Items #1 (“I have difficulty getting work done”) and #7 (“I have difficulty thinking about anything else”) both pertain to difficulties with concentration and directing cognitive efforts toward desired goals. Items #3 (“I feel ashamed with myself for feeling that way”) and #6 (“I become irritated with myself for feeling that way”) both address secondary emotional reactions (i.e., self-directed shame and irritability) stemming from the non-acceptance of one’s unwanted or negative emotions. These content and semantic similarities suggest a potential interdependence between these pairs of items. In other words, if an individual scores high on one item, it is very likely that they will also score high on the corresponding item, thereby creating an interdependent relationship that calls for correlating the residuals. The two items that were not correlated are item #5 (“I believe that there is nothing I can do to feel better”) and item #8 (“It takes me a long time to feel better”). In this case, no potential interdependence is evident; indeed, someone who believes there is nothing they can do to feel better does not necessarily take a long time to actually feel better. Furthermore, in Penner’s original DERS-8, Item Response Theory (IRT) was used, and the issue of correlating residuals did not arise because IRT employs different fit indices than those used in CFA, based on distinct measurement theories.

Regarding convergent-divergent validity, in Study 1 DERS-8 shows very strong positive associations with difficulties in identifying emotions – a core feature of alexithymia – while it is less correlated with the ERQ dimensions of cognitive reappraisal and emotion suppression. Additionally, DERS-8 exhibits a moderate correlation with the alexithymia subscale for describing and communicating emotions, whereas it does not correlate with EOT (externally oriented thinking) of the TAS-20. Finally, DERS-8 is positively correlated with impulsivity, highlighting the deep relationship among these constructs despite their differences [122–124]. Concerning the psychometric characteristics, the IT-DERS-8 demonstrated strong reliability and internal consistency. To note, ERD can be conceptualized through different theoretical frameworks, leading to distinct measurement approaches. The DERS is grounded in a multidimensional model that views ERD as deficits across several core domains, including emotional awareness, clarity, acceptance, and access to effective regulation strategies [7]. In contrast, the Emotion Regulation Questionnaire (ERQ) [84] adopts a process-focused approach, rooted in Gross’s [83] process model of emotion regulation, which distinguishes between antecedent-focused and response-focused approaches specifically measuring the habitual use of two discrete strategies: cognitive reappraisal and expressive suppression. These divergent theoretical foundations may explain the modest correlations typically observed between these instruments, as they capture different aspects of the broader emotion regulation construct.

### Study 2

In Study 2, using an independent sample twice the size of that in Study 1, the results of the CFA were first replicated. In this instance too, without correlating the residuals, the measurement model exhibited an unacceptable fit due to the RMSEA. Subsequently, by correlating the same pairs of residuals as in Study 1, the model achieved a good fit. This demonstrates that in independent samples there is the same need to correlate the residuals of certain pairs of items that are linked both semantically and in terms of content. Therefore, these correlations were not an isolated finding from the first study but likely indicate that the DERS-8 scale itself requires these modifications to the model. The structural validity of the IT-DERS-8 was once more validated, demonstrating good fit indices to the data. Each item was associated with the proposed latent factor, showing a robust relationship that suggests they effectively represent the construct [93, 125].

In Study 2, the correlation analysis was consistent with that of Study 1, showing that independent large sample reported similar levels of association with related constructs, suggesting the robustness of the tool and its convergent validity. Nonetheless, the pattern of correlations with related constructs reveals a nuanced picture. Moderate correlations with the DIF and DDF subscales of the TAS-20 indicate that the IT-DERS-8 effectively taps into core aspects of emotional awareness and expression difficulties. However, smaller correlations with the EOT subscale and with cognitive reappraisal and emotional suppression suggest that the IT-DERS-8 captures a somewhat distinct, more focused dimension of ERD rather than broadly overlapping with all aspects of related emotional constructs. This variability in correlation magnitudes aligns with theoretical expectations, given that ERD are multidimensional and not entirely reducible to alexithymia facets or specific regulation strategies. The smaller associations also highlight that the IT-DERS-8 does not simply replicate other measures but offers unique, complementary insights into emotion regulation processes.

The measurement invariance analysis revealed that the DERS-8 is invariant across gender and age up to the latent means – a finding that replicates the results obtained with the DERS-SF [56]. Evaluating measurement invariance (MI) across diverse populations is essential for validating a scale. Previous research has identified age as a key predictor of emotion regulation, with older individuals typically exhibiting better regulation [56, 126, 127]. In Study 2, the MI analysis indicates that the IT-DERS-8 is invariant across all examined groups, including different age cohorts (younger than 49 years versus 50 years and older) and between genders (men and women). Specifically, the scale achieved strict invariance—meaning that, at equivalent levels of the latent trait, the expected item responses were consistent across groups—as well as latent mean invariance, with the compared groups showing similar expected latent means. These findings highlight the effectiveness and reliability of the IT-DERS-8 in measuring the intended construct independently of age and gender. As a result, DERS-8 can be confidently utilized to compare outcomes across various gender and age groups, as it preserves a consistent factorial structure, equivalent item-factor relationships, and stable item thresholds. Measurement invariance of the IT-DERS-8 across sex and age groups supports that the scale assesses ERD consistently across these demographics, which is an important psychometric prerequisite for its use in diverse populations. However, while these findings strengthen the scale’s measurement equivalence, further research is needed to establish its clinical utility, including validation within clinical samples and examination of its sensitivity to clinical interventions.

Regarding cut-off points, since no gold standards have been established in the literature for differentiating levels of ERD from non-problematic to at-risk, a procedure previously employed in earlier studies was used [95, 96] – despite more accurate procedures certainly exist to define them. Also, we acknowledge that this approach relies on factor scores and the potential issues they may entail [128], the particularly large sample size (*N* = 4221) makes the results highly reliable, especially given that the test is invariant across gender and age. The cut-off definition is useful but one should be aware that it is a statistically driven procedure. Moreover, it should be noted that to determine the cut-offs were used only specific variables that demonstrated clinical relevance and stronger convergent validity with ERD. As instance, the decision not to use the EOT was driven by its conceptual divergence as it reflects a more cognitive style of alexithymia less directly related to emotional regulation processes, its diagnostic utility for differentiating levels of ERD is limited. Indeed, it also has relatively weak association with core ERD measured by IT-DERS-8. Similarly, impulsivity was not measured in Study 2 to determine the cut-offs because, as assessed by the BIS-11, it reflects a broad, trait-like tendency toward rapid and unplanned actions, encompassing attentional, motor, and non-planning dimensions. This general conceptualization of impulsivity differs from the more specific emotional processing and regulatory challenges measured by the IT-DERS-8, which focuses on difficulties managing emotions rather than generalized impulsive traits. Although impulsivity and ERD are related constructs, including the BIS-11 in ROC cut-off determination for ERD could reduce the specificity of these thresholds. Therefore, to enhance the clinical relevance and interpretability of cut-offs, we prioritized measures directly aligned with core emotion regulation deficits.

It is important to highlight that the ROC curve showed a moderate/high effect size with the chosen independent variable (AUC = 0.864). Furthermore, it is worth noting that the identified cut-off point (IT-DERS-8 *≥* 25) demonstrates both moderate SE (crucial for identifying “positive” cases) and strong SP (essential for detecting “negative” cases). However, it is important to emphasize that a cut-off point like the one found cannot definitively distinguish “healthy” individuals from “pathological” ones. In fact, the DERS and its shorter versions (DERS-SF and DERS-8) were not primarily conceptualized as screening tests but rather as tools for measuring a construct that exists on a continuum [13, 55]. It is also worth remembering that this construct is transdiagnostic and a common trait in the general population [4, 55]. In this case, the strong SP associated with the cut-off point appears suitable for identifying as “at risk” only those individuals with extreme IT-DERS-8 scores – indeed, out of 4221 subjects, only 13.31% appear to be potentially problematic (and therefore might require further clinical assessment). Lastly, it is important to note that the cut-off point of 25 is associated with particularly satisfactory ACC (= 0.879). This result suggests that this threshold is capable of accurately distinguishing “true” cases from “false” ones, as the misclassification rate (12.09%) is small compared to the correctly classified cases (87.92%).

### Limitations and future research

This research has several limitations that also suggest directions for future research. Both studies relied on online convenience snowball sampling of the general population, a common method when exploring transdiagnostic constructs such as ERD. Snowball sampling through social media relies on participants recruiting others within their social networks and can introduce sampling biases because of overrepresentation of certain demographic groups who are more active online, while underrepresenting less connected or offline populations. The cut-off, despite being derived through a well-established procedure, is based on a statistical method without clinical judgment. However, caution is needed when applying these findings to clinical populations, who may function at lower levels than those not seeking treatment. Despite this, future cross-cultural research is expected to independently evaluate, validate and confirm the scale’s psychometric characteristics and factor structure across diverse countries, languages, populations (including clinical and non-clinical groups), and age ranges (such as adolescents).

The sample comprised a large cohort of participants spanning a wide age range (18 to 84 years), which is particularly valuable given that ERD manifests across the lifespan [30, 126, 127]. However, the sample was predominantly female (79.4%), resulting in an unbalanced sex distribution that limits the representativeness of the findings. This demographic imbalance should be considered when interpreting the results, as it may constrain the generalizability to male populations. To address this limitation, future research should aim for more balanced recruitment to better explore potential sex-related differences in ERD. Moreover, the exclusive use of self-report measures and the cross-sectional design limited our ability to examine changes over time and assess predictive validity (e.g., test-retest reliability and longitudinal measurement invariance). These results are generalizable to the adult Italian general population, future studies will consider adolescents and clinical samples. Future studies might address these issues by identifying recurring ERD patterns through the development of latent psychological profiles. While the DERS-8 offers the advantage of brevity and maintains good psychometric properties, it is important to acknowledge that, compared to the original DERS-36, it does not include the subscales Awareness and Clarity that tap into emotional awareness and understanding. This reduction may result in some loss of conceptual breadth, potentially limiting the tool’s ability to fully capture the multifaceted nature of emotion regulation deficits. Although the IT-DERS-8 remains a valid and practical instrument for screening and research purposes, future studies should directly compare its performance with the full DERS-36 to evaluate how well the shorter version captures the complete construct of ERD, particularly in clinical contexts where a more detailed assessment might be necessary.

Although the IT-DERS-8 shows good psychometric properties and initial sensitivity, its thresholds have not yet been validated against clinical diagnostic criteria or outcomes. Although the multi-step ROC-based procedure for cut-off estimation is methodologically sound, it lacks direct clinical anchoring through the use of diagnostically confirmed samples or symptom-based grouping via structured interviews. Incorporating clinically diagnosed participants or validated symptom measures for depression or anxiety would enhance the ecological validity and clinical applicability of identified thresholds. Therefore, the proposed cut-offs should be considered preliminary and require further validation in clinical studies with rigorously characterized samples to establish their utility in clinical assessment and decision-making. Therefore, the IT-DERS-8 use for clinical evaluation has to be established by further research about criterion validity, test-retest reliability, predictive utility, longitudinal predictive validity, and sensitivity to clinical interventions. Future psychometric evaluations in clinical populations are advocated.

Lastly, it is crucial to emphasize that this cut-off is not diagnostic and should not be used for clinical screening purposes until validated against structured clinical interviews or established symptom thresholds in clinical samples.

### Strengths

Despite its limitations, this research offers several important strengths from both methodological and clinical perspectives. It is the first to validate the IT-DERS-8, providing a precise yet efficient way to assess this widely recognized construct. DERS-8 reduces administration time and participant burden, making it especially useful for screening or in extensive assessment batteries in both clinical and research settings. The two studies conducted in two large independent samples exactly replicated the same results, supporting their robustness. The whole research relied on strong methodological design and sound statistical analysis in line with scientific guidelines. Moreover, the normative scores established for the IT-DERS-8 enable practitioners to readily determine an individual’s standing within a distribution. Additionally, the IT-DERS-8 demonstrates strong psychometric properties also because it excludes the controversial Awareness and Clarity subscales, which have been consistently shown to have weak reliability and low relevance to core ERD [40, 60]. By focusing on more central aspects of emotion regulation, the DERS-8 provides a more valid and reliable assessment tool, aligning with findings from previous research on shortened DERS versions.

Findings from the present research are consistent with other validations of the DERS-8 worldwide, including the original validation by Penner [66] among adolescents with psychiatric disorders, the study by Zhang in Chinese adolescents [72], and the research by Larionow in a large Polish adult sample [67]. All of these studies identified a unidimensional factorial structure, demonstrated good reliability, and confirmed strong psychometric properties.

Notably, DERS-8 had not been validated in the Italian context previously, and its longer versions pose challenges for inclusion in comprehensive assessment batteries. By providing the first validation of the DERS-8 in Italian, this study makes a significant incremental contribution enabling researchers and clinicians to utilize it with individuals speaking Italian.

### Implications for clinical and research practice

The IT-DERS-8 offers substantial clinical utility as an assessment instrument, particularly because ERD are a transdiagnostic factor found across various disorders and psychological challenges of differing severity [9]. ERD plays multiple role, it can trigger, result from, or perpetuate psychological distress [129, 130], thereby providing critical insights for both conceptualizing and treating clinical conditions. Regarding future directions, the IT-DERS-8 helps clarify psychological challenges and may contribute to inform the tailoring of specific interventions, such as Dialectical Behavioral Therapy. Its brevity makes it especially valuable in settings where patients—like those with organic illnesses or younger individuals—might otherwise experience assessment fatigue or boredom. Researchers and clinicians can choose the DERS-8 when a brief, general index of ERD is sufficient, acknowledging that some nuances of emotional awareness and clarity may be lost, or choose the DERS-SF when a more detailed, facet-specific emotion regulation profile is desired. In terms of research implications within clinical contexts, future studies could benefit from examining the IT-DERS-8’s characteristics and associations in targeted populations (e.g., trauma survivors, individuals with health-related issues, personality disorders, or eating disorders) [e.g., 131–133]. In this regard, network analysis presents a promising method to determine whether the item configurations remain consistent across different groups [134, 135].

## Conclusions

In summary, the findings indicate that the IT-DERS-8 exhibits strong construct validity and reliability. Consequently, the IT-DERS-8 serves as a dependable and valid self-report instrument for assessing ERD in adults, regardless of age or gender. This tool can be instrumental in designing interventions aimed at enhancing psychological well-being.

## Supplementary Information

Below is the link to the electronic supplementary material.

Supplementary Material 1

Supplementary Material 2

## Data Availability

The datasets presented in this article are not readily available because due to privacy restrictions, data were available from the corresponding author on a reasonable request.
