# Supplementary material for: The Italian Version of the Difficulties in Emotion Regulation Scale-8 (DERS-8): A Two-Step Assessment of Structural Validity, Psychometric Properties, and Clinical Cut-Off
Source: BMC Psychol. 2025 Oct 14;13:1137. doi: 10.1186/s40359-025-03482-6 (PMC12522448; doi:10.1186/s40359-025-03482-6)
Supplement: Supplementary file 1 — Supplementary Material 1 [file 40359_2025_3482_MOESM1_ESM.pdf]

DERS-8

ISTRUZIONI:

Questo questionario contiene una serie di atteggiamenti **relative a come le persone sono in relazione con i loro sentimenti e/o emozioni** di cui possono fare esperienza.

Legga attentamente ogni frase e poi **segni** – nella casella corrispondente – ***quanto sente che quell'atteggiamento la rispecchia nella quotidianità***.

NON ESISTONO RISPOSTE GIUSTE O SBAGLIATE

1	2	3	4	5
QUASI MAI	A VOLTE	LA METÀ DELLE VOLTE	MOLTE VOLTE	QUASI SEMPRE

**1**	Quando sono turbato, ho delle difficoltà a completare il mio lavoro	**1**	**2**	**3**	**4**	**5**
**2**	Quando sono turbato, perdo il controllo	**1**	**2**	**3**	**4**	**5**
**3**	Quando sono turbato, mi vergogno di me stesso perché mi sento in quel modo	**1**	**2**	**3**	**4**	**5**
**4**	Quando sono turbato, ho delle difficoltà nel controllare i miei comportamenti	**1**	**2**	**3**	**4**	**5**
**5**	Quando sono turbato, credo che non ci sia niente che io possa fare per sentirmi meglio	**1**	**2**	**3**	**4**	**5**
**6**	Quando sono turbato, mi irrito con me stesso perché mi sento in quel modo	**1**	**2**	**3**	**4**	**5**
**7**	Quando sono turbato, faccio fatica a focalizzarmi su altre cose	**1**	**2**	**3**	**4**	**5**
**8**	Quando sono turbato, mi ci vuole molto tempo per sentirmi meglio	**1**	**2**	**3**	**4**	**5**

Rossi, AA, Panzeri, A, & Mannarini, S. (2025). The Italian Version of the Difficulties in Emotion Regulation Scale-8 (DERS-8): A Two-Step Assessment of Structural Validity, Psychometric Properties and Clinical Cut-Off. *BMC Psychology.*
