# Supplementary material for: The Italian Version of the Difficulties in Emotion Regulation Scale-8 (DERS-8): A Two-Step Assessment of Structural Validity, Psychometric Properties, and Clinical Cut-Off
Source: BMC Psychol. 2025 Oct 14;13:1137. doi: 10.1186/s40359-025-03482-6 (PMC12522448; doi:10.1186/s40359-025-03482-6)
Supplement: Supplementary file 2 — Supplementary Material 2 [file 40359_2025_3482_MOESM2_ESM.pdf]

The Italian Version of the Difficulties in Emotion Regulation Scale 8 (DERS-8): A Two-Step Assessing Structural Validity, Psychometric Properties and Clinical Cut-Off.

Alessandro Alberto Rossi*, Anna Panzeri, Stefania Mannarini

SUPPLEMENTARY 2

Corresponding author
Alessandro Alberto Rossi
Department of Philosophy, Sociology, Education, and Applied Psychology, Section of Applied Psychology, University of Padova, Padova, Italy.
Via Venezia 12, 35131, Padua (PD), Italy
Email: a.rossi@unipd.it

Table S1. Comparison between the items of the original English version and those of the Italian version extracted from previous Italian translations of the DERS.

Original	Italian
When I'm upset, I have difficulty getting work done.	Quando sono turbato, ho delle difficoltà a completare il mio lavoro
When I'm upset, I feel out of control.	Quando sono turbato, perdo il controllo
When I'm upset, I feel ashamed with myself for feeling that way.	Quando sono turbato, mi vergogno di me stesso perché mi sento in quel modo
When I'm upset, I have difficulty controlling my behaviors.	Quando sono turbato, ho delle difficoltà nel controllare i miei comportamenti
When I'm upset, I believe that there is nothing I can do to ... feel better.	Quando sono turbato, credo che non ci sia niente che io possa fare per sentirmi meglio
When I'm upset, I become irritated with myself for feeling that way.	Quando sono turbato, mi irrito con me stesso perché mi sento in quel modo
When I'm upset, I have difficulty thinking about anything else.	Quando sono turbato, faccio fatica a focalizzarmi su altre cose
When I'm upset, It takes me a long time to feel better	Quando sono turbato, mi ci vuole molto tempo per sentirmi meglio

Table S2. Sample of Study 1 *plus* Study 2. Raw scores, normative scores, and percentile distribution of the IT-DERS-8.

Norm score table (N = 6237)		
Raw	T-score	Percentile
8	27.8	1.300
9	31.1	2.900
10	35.1	6.800
11	39.1	13.800
12	42.4	22.300
13	45.0	30.700
14	47.1	38.600
15	49.0	45.900
16	50.6	52.500
17	52.1	58.500
18	53.5	63.800
19	54.8	68.600
20	56.1	72.800
21	57.2	76.500
22	58.4	79.900
23	59.5	82.800
24	60.5	85.400
25	61.6	87.600
26	62.6	89.600
27	63.6	91.300
28	64.6	92.800
29	65.6	94.100
30	66.6	95.200
31	67.6	96.100
32	68.7	96.900
33	69.7	97.600
34	70.8	98.100
35	72.0	98.600
36	73.3	99.000
37	74.7	99.300
38	76.5	99.600
39	79.6	99.800
40	80.0	99.900
